# Whey Protein Peptides Have Dual Functions: Bioactivity and Emulsifiers in Oil-In-Water Nanoemulsion

**DOI:** 10.3390/foods11121812

**Published:** 2022-06-20

**Authors:** Randy Adjonu, Gregory S. Doran, Peter Torley, Gilbert O. Sampson, Samson O. Agboola

**Affiliations:** 1 School of Dentistry and Medical Science, Charles Sturt University, Wagga Wagga, NSW 2678, Australia; 2 The Gulbali Institute, Charles Sturt University, Albert Pugsley Place, Wagga Wagga, NSW 2678, Australia; gdoran@csu.edu.au (G.S.D.); sagboola@csu.edu.au (S.O.A.); 3 School of Agricultural, Environmental and Veterinary Sciences, Charles Sturt University, Wagga Wagga, NSW 2678, Australia; 4 School of Science, RMIT University, Bundoora, VIC 3083, Australia; peter.torley@rmit.edu.au; 5 Faculty of Vocational Education, University of Education Winneba, Kumasi Campus, Kumasi P.O. Box 1277, Ghana; gosampson@uew.edu.gh

**Keywords:** ultrafiltration, nanoemulsions, droplet size, zeta potential, bioactive peptide, storage stability

## Abstract

Whey protein isolate (WPI)-derived bioactive peptide fractions (1–3, 3–5, 5–10, 1–10, and >10 kDa) were for the first time used as emulsifiers in nanoemulsions. The formation and storage stability of WPI bioactive peptide-stabilized nanoemulsions depended on the peptide size, enzyme type, peptide concentration, and storage temperature. The highly bioactive <10 kDa fractions were either poorly surface-active or weak stabilizers in nanoemulsions. The moderately bioactive >10 kDa fractions formed stable nanoemulsions (diameter = 174–196 nm); however, their performance was dependent on the peptide concentration (1–4%) and enzyme type. Overall, nanoemulsions exhibited better storage stability (less droplet growth and creaming) when stored at lower (4 °C) than at higher (25 °C) temperatures. This study has shown that by optimizing peptide size using ultrafiltration, enzyme type and emulsification conditions (emulsifier concentration and storage conditions), stable nanoemulsions can be produced using WPI-derived bioactive peptides, demonstrating the dual-functionality of WPI peptides.

## 1. Introduction

Whey proteins and their hydrolysates are important emulsifiers in food emulsions [1,2,3,4] and are also sources of significant bioactive peptide molecules [5,6], which are beneficial for promoting good health in humans. Whey protein peptides have antioxidant, angiotensin-I-converting enzyme (ACE)-inhibition and anti-hypertensive [5,7], opiate [7], immunomodulatory [8], and antidiabetic [9] properties. These peptides are considered safe and healthy compounds [7], easily absorbed by the human body, and used as functional and nutraceutical agents.

The utilization of bioactive peptides in foods requires the design of suitable food matrices, capable of protecting and delivering bioactive peptides in foods, while minimizing interactions with other food components [3]. Moreover, nanoemulsion systems are excellent for delivering bioactive compounds in foods and beverages [10,11]. Oil-in-water nanoemulsions have distinct physicochemical properties from conventional emulsions, including smaller droplet sizes, a larger surface area, enhanced stability, distinctive interfacial and bulk rheological properties, and scatter light weakly [12]. These characteristics make nanoemulsions ideal systems for incorporating bioactive compounds into foods, beverages, and gels [4,12,13]. Nanoemulsions also aid in the transport and controlled delivery, encapsulation, bioavailability and bioaccessibility of lipophilic and phytophenolic food components, such as β-carotene, vitamin E, polyunsaturated fatty acids, essential oils, and antimicrobial compounds [12,13,14,15]. Moreover, nanoencapsulation of bioactive peptides provides a pathway for including bioactive peptides in foods.

Alternatively, bioactive peptides may be applied as emulsifiers in food or pharmaceutical nanoemulsions. Protein hydrolysates are effective emulsifiers in conventional food emulsions [16,17,18], and whey protein isolate (WPI) hydrolysates are effective emulsifiers in food nanoemulsions [1]. When using only crude WPI hydrolysates, the formation and stability of nanoemulsions depended on the enzyme type, dispersed phase volume, and peptide emulsifier concentrations. Despite their demonstrated efficacy in conventional emulsions, there are limited studies on using protein hydrolysates and their peptide fractions as emulsifiers in nanoemulsions.

The current work investigated the emulsifying properties of WPI bioactive peptidic fractions in nanoemulsions. Using WPI-derived bioactive peptides as emulsifiers in nanoemulsions may increase the incorporation of bioactive peptides in food systems and lead to products with modified texture, taste, and sensory attributes. The study had two objectives; first, to investigate the emulsifying ability of WPI-derived bioactive peptide fractions as emulsifiers in nanoemulsions, demonstrating their dual functionality in food systems. Secondly, to study the effects of peptide concentration, storage conditions, and peptide fractionation on the stability of WPI bioactive peptide-stabilized nanoemulsions.

## 2. Materials and Methods

### 2.1. Materials

Whey protein isolate (WPI) was purchased from MyoPure Pty Ltd., Petersham, Australia, and canola oil was obtained from a local supermarket. α-Chymotrypsin, pepsin, Tween 40, 6-hydroxy-2,5,7,8-tetramethylchroman-2-acid, and 2,2′-azinobis-(3-ethylbenzothiazoline-6-sulfonic acid) diammonium salt (ABTS) were purchased from Sigma-Aldrich (Sydney, Australia). All other chemicals were of reagent grade. Concentrations are calculated on a *w*/*v* basis unless otherwise stated.

### 2.2. Preparation of WPI Bioactive Peptide Fractions

Hydrolysis was carried out as previously described [6]. Briefly, WPI (5% *w*/*v*) was suspended in 10 mM phosphate buffer, pH 7.00. The suspension was adjusted to pH 2.60 for pepsin or 7.80 for chymotrypsin hydrolysis. Enzymes were then added at an enzyme: substrate ratio of 1:40 and the suspensions were incubated at 37 °C for 3 h with constant stirring. After the hydrolysis, the enzymes were inactivated by heating the hydrolysates at 90 °C for 15 min. The crude hydrolysates were subsequently fractionated by ultrafiltration (UF). Ultrafiltration was carried out in a Model 8200 Amicon Stirred Ultrafiltration cell, fitted with 1, 3, 5, and 10 kDa molecular weight (MW) cut-off cellulose membranes (Millipore Australia Pty Ltd., Melbourne, Australia), under nitrogen gas. Ultrafiltration was performed sequentially and four peptide fractions (1–3, 3–5, 5–10, and >10 kDa) were collected, concentrated by rotary evaporation, and freeze-dried. For this discussion, the >10 kDa fractions are labelled as UC–10F and UP–10F for the chymotrypsin and pepsin fractions, respectively. The designations, 1–3, 3–5, 5–10 kDa, are unchanged for the other fractions.

### 2.3. Bioactivity and Hydrophobicity Measurement

The fractions were assessed for their free radical scavenging antioxidant (µmol TE/mg peptide) and ACE-inhibition activities (IC_50_), and surface hydrophobicity (So), using the anionic fluorescence probe, 8-anilino-naphthalene-1-sulfonate (ANS). Details of these assays are described in Adjonu et al. [6].

### 2.4. Formation of WPI Bioactive Peptide-Stabilized Nanoemulsions

Nanoemulsions were prepared by homogenizing 4% oil and 96% aqueous phase [6]. The aqueous phase contained either 2% of the small to medium size (1–3, 3–5, 5–10 kDa) or 1% of the larger size (>10 kDa) bioactive peptide as emulsifiers. The aqueous phase was 10 mM phosphate buffer (pH 7.00, containing 0.02% sodium azide). Peptide concentrations were chosen based on their MW size ranges. Peptide solutions were stirred for 1 h at room temperature to ensure complete hydration. A coarse premix emulsion was first prepared by homogenizing the oil and aqueous phase, using an Ultra Turrax (T25 basic, Janke & Kunkel IKA Labortechnik, Staufen, Germany) for 4 min. Nanoemulsions were formed by passing the coarse emulsions through a high-pressure homogenizer (EmulsiFlex-C5, Avestin, Inc., Ottawa, ON, Canada), using four passes at 100 to 150 MPa. Nanoemulsions were then separated into aliquots and stored at 4 °C or 25 °C for 7 d. To study the effect of peptide concentration on nanoemulsion formation, additional nanoemulsions were formed with fractions UC–10F and UP–10F at 2 and 4%. A nanoemulsion was also produced with the low MW non-ionic surfactant Tween 40 (1%) for comparison purposes.

### 2.5. Droplet Size, Size Distribution, and Zeta (ζ) Potential Measurement

The size (diameter) and distribution of (nano)emulsion droplets were measured by dynamic light scattering using a Zetasizer Nano ZS (Malvern Instruments, Worcester, UK). Measurements were performed at 25 °C with duplicate determinations for each sample. Before measurement, the (nano)emulsions were diluted in phosphate buffer (10 mM, pH 7.00) at a dilution ratio of 1:100.

The ζ-potential of the (nano)emulsions was measured using a universal ‘Dip cell’ (ZEN1002, Malvern Instruments, Worcester, UK) on the Zetasizer Nano ZS. (Nano)emulsions were diluted (1:50) in the phosphate buffer and then equilibrated at 25 °C for 60 sec before measurement. The ζ-potential was also measured for the 1%, 2%, and 4% aqueous peptide solutions. Duplicate determinations were performed on each sample.

### 2.6. Storage Studies

The stability of the (nano)emulsions was determined by monitoring changes in droplet size, size distribution, ζ-potential, and cream development over 7 d of storage at 4 °C and 25 °C [6]. Creaming was determined by placing the (nano)emulsions (5 mL) into 6 mL sterile syringes. After the appropriate storage time, the volume of the cream layer was read off the graduations on the syringes, and the creaming index (%) was calculated.

### 2.7. Statistical Analysis

All experiments were performed in duplicate, and results were reported as the mean ± standard deviation. Analysis of variance was performed on the bioactivity and surface hydrophobicity data (Statgraphics^®^ Centurion XVI, StatPoint Technologies, Inc., Warrenton, VA, USA) and mean separation was performed using Fisher’s least significant difference (LSD) test (*p* < 0.05).

## 3. Results and Discussion

### 3.1. Bioactivity of WPI Peptide Fractions

All WPI peptide fractions showed antioxidant activity, influenced by the peptide size and the enzyme used (Table 1). The 1–3 kDa pepsin fraction had higher antioxidant activity than the fraction UP–10F (*p* < 0.05). No other mean comparisons were significantly different. Fractions UC–10F and UP–10F had lower antioxidant activity than the <10 kDa fractions; however, the activities were about 63 to 86% of the average activities of the <10 kDa fractions. Except for the fraction UP–10F, the antioxidant activities were comparable to those reported for unfractionated WPI hydrolysates [6]. The size range of bioactive peptides varies and includes peptides with MW less or greater than 10 kDa [19].

Whey protein peptides can inhibit the activity of ACE [5]. To complement the antioxidant activity, WPI peptides were screened for their ACE-inhibition activity. The IC_50_ values for ACE-inhibition of peptides are also presented in Table 1. The 1–3 kDa fractions had significantly higher ACE-inhibition activities than the other fractions, with the 1–3 kDa pepsin fraction showing the greatest potency (*p* < 0.05). Fraction UC–10F exhibited greater ACE-inhibition than the fraction UP–10F (*p* < 0.05). Like the antioxidant activity, the smaller to medium size <10 kDa fractions possessed greater inhibitory potencies than larger size >10 kDa fractions, which is consistent with previous studies [20,21]. Moreover, the IC_50_ values for the peptide fractions were comparable to those reported by da Costa et al. [20]. Overall, the <10 kDa fractions exhibited higher bioactivities, while only moderate bioactivities were observed for the >10 kDa fractions, regardless of enzyme choice. Thus, UF enriched different bioactive peptides in specific fractions with the prospect of being applied as functional ingredients in functional foods.

### 3.2. Surface Hydrophobicity of WPI Peptide Fractions

The surface hydrophobicity of proteins is a useful indicator of protein/peptide conformational changes, a relative measure of peptide surface activity, and relates the protein/peptide structure to its emulsifying property [16,22]. Compared to the fractions UC–10F and UP–10F, the <10 kDa fractions exhibited significantly lower surface hydrophobicity values (*p* < 0.05) (Table 1). The <10 kDa peptides had fewer hydrophobic pockets or ANS probe-specific binding sites, which is consistent with previous reports on low MW peptides from soy protein isolate [23] and whey protein [16] hydrolysates. Hydrolysis increased the molecular charge of the smaller size peptides contained in the <10 kDa fractions, decreasing their hydrophobic properties [16].

Fraction UP–10F had more hydrophobic peptides than fraction UC–10F, as shown by the significantly larger surface hydrophobicity value (*p* < 0.05) (Table 1). Differences observed between these two fractions likely resulted from the different peptide bond specificities of the chymotrypsin and pepsin enzymes. Chymotrypsin hydrolyses the C-terminal end, whereas pepsin hydrolyses the N-terminal end of aromatic and hydrophobic amino acids. The actions of these enzymes may lead to exposure of specific peptide sequences with greater affinity to bind to the ANS probe, and hence a higher surface hydrophobicity, as demonstrated by the fraction UP–10F. The surface hydrophobicity values of fractions UC–10F and UP–10F were significantly larger than the values reported for unfractionated WPI hydrolysates and unhydrolyzed WPI (647–809) [6]. Thus, enzymatic hydrolysis and UF enriched larger and more hydrophobic peptides from the crude WPI hydrolysates, which are vital to their interfacial and emulsifying properties in nanoemulsions.

### 3.3. Effect of WPI Peptide Size on Nanoemulsion Formation

#### 3.3.1. Less than 10 kDa (1–3, 3–5, 5–10 kDa) Fractions

Emulsions containing 2% peptide emulsifiers were produced with the 1–3, 3–5, and 5–10 kDa WPI fractions. None of the pepsin WPI fractions formed nanoemulsions; diameter = 1233–4122 nm (Table 2, 0 h). The droplet size of these emulsions decreased after 12 h of storage at 25 °C. Conversely, all the chymotrypsin WPI fractions formed nanoemulsions (diameter = 199–290 nm); however, these were only transiently stable and exhibited extensive droplet growth (Table 2), with phase separation (Supplementary Figure S1) after 12 h of storage. Similar results were reported by Schröder et al. [16], Euston et al. [24], Agboola et al. [25], and Mutilangi et al. [26] for whey protein hydrolysate/peptide-stabilized conventional emulsions, with such emulsions exhibiting poor stability.

The magnitude of the ζ-potentials was greater for the chymotrypsin fraction nanoemulsions than the pepsin fraction emulsions (Table 2). Regardless of the enzyme type, the magnitude of the ζ-potentials decreased (i.e., less negative) as the size range of the peptides increased. The ζ-potentials decreased for the chymotrypsin fraction nanoemulsions but tended to increase for the pepsin fraction emulsion after 12 h of storage. However, the changes in droplet size and ζ-potentials were not correlated. Compared to the small MW non-ionic surfactant, Tween 40 (Table 2, diameter = 166 nm), the <10 kDa WPI bioactive fractions were either poorly surface-active (pepsin fractions) or weak stabilizers (chymotrypsin fractions) in nanoemulsions.

#### 3.3.2. Greater than 10 kDa (UC–10F and UP–10F) Fractions

As shown in Table 2, fractions UC–10F and UP–10F formed nanoemulsions: diameter = 196 nm and 174 nm, and ζ-potentials = −28.3 mV and −33.2 mV for UC–10F and UP–10F, respectively. The droplet sizes were slightly larger than Tween 40 stabilized nanoemulsions (Table 2). These nanoemulsions were stable against creaming and phase separation after 24 h of storage at 25 °C, emphasizing the importance of peptide size to the interfacial and stabilizing properties [16] of WPI bioactive peptides in nanoemulsions. However, the UC–10F and UP–10F nanoemulsion droplet sizes increased slightly after 24 h, due to the rearrangement and re-alignment of peptides at the droplet interface [16].

Emulsion formation and stability are two separate processes, governed by different properties of the peptide emulsifier. Emulsion formation is governed by adsorption behavior (diffusivity and the interfacial property), whereas stability depends on the size and proportion of larger peptides present [16]. Likewise, proteins stabilize emulsion droplets by electrostatic and steric effects, the formation of viscoelastic films, and modifications of the bulk rheology [22,27]. The <10 kDa pepsin WPI fractions had poor interfacial activity to form and stabilize nanoemulsion droplets. Thus, most of the peptides saturated the aqueous phase, preventing their accumulation at the oil–water interface to reduce the interfacial tension, hence, leaving a proportion of the oil phase unhomogenized [22,24]. Although the <10 kDa WPI chymotrypsin fractions formed nanodroplets (Table 2), they only adsorbed weakly to the droplet interface to form weak interfacial films [16,27]. In their work, Agboola et al. [25] emphasized that large MW peptides are critical to the formation and stability of whey protein hydrolysate-stabilized emulsions. The <10 kDa WPI fractions were deficient in a diverse range of larger-sized peptides and lacked the structure and conformational freedom to stabilize the droplets [22,25,28]. Consequently, the thickness and rheology of the adsorbed peptide layer were reduced, which escalated droplet coalescence and flocculation [24]. Moreover, the <10 kDa peptide (nano)emulsions had high ζ-potentials, suggesting that emulsion instability resulted from structural and conformational factors (e.g., diminished peptide size) and viscous effects rather than electrostatic effects. The mechanism of destabilization is illustrated briefly in Supplementary Figure S2.

Fractions UC–10F and UP–10F formed stable nanoemulsions because they contained a more diverse range of larger and surface-active peptides [29], capable of forming and stabilizing nanoemulsion droplets. Fractions UC–10F and UP–10F constituted about 22 to 46% of the total peptide yield recovered after UF treatment. The >10 kDa fractions were rich in larger MW peptides and some intact protein materials with charge hydrophobic domains [30] and accounted for the significantly high surface hydrophobicity values (Table 1). These culminated in better interfacial activity, stronger and cohesive viscoelastic films, and electrostatic stabilization in nanoemulsions [16,31]. Since the <10 kDa bioactive WPI fractions acted poorly as stabilizers in nanoemulsions, only fractions UC–10F and UP–10F are discussed in subsequent sections.

### 3.4. Effect of WPI Bioactive Peptide Concentration on (Nano)Emulsion Formation

**Figure 1:**
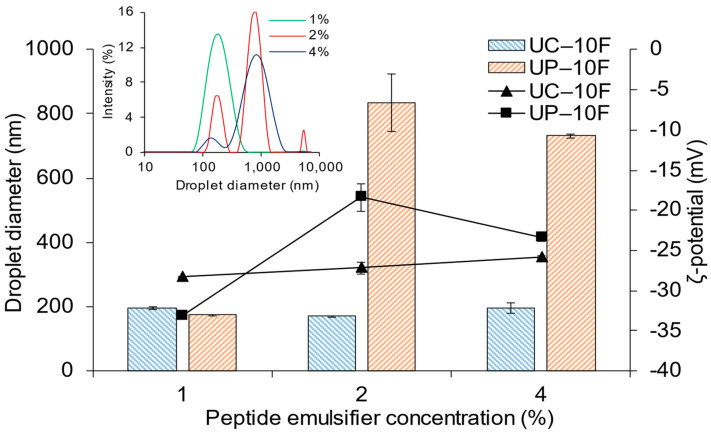
Effect of peptide concentration on the droplet diameter and ζ-potential of nanoemulsions or emulsions formed with the UC–10F chymotrypsin and UP–10F pepsin WPI fractions. Legend: droplet size, bars; ζ-potential, lines. Insert, upper left: droplet size distribution of UP–10F (nano)emulsions at 1, 2, and 4% emulsifier concentrations.

Varying the concentration (1 to 4%) of fractions UC–10F and UP–10F had different effects on the (nano)emulsions. Droplet sizes were larger at the 2 and 4% UP–10F concentrations than at 1% but remained relatively constant over the range of concentrations for the UC–10F nanoemulsions (Figure 1, bars, left axis). The droplet size distribution was monomodal in the 1% UP–10F nanoemulsion only (Figure 1, insert—upper left) but was monomodal at all UC–10F concentrations. Visually, the 2 and 4% UP–10F emulsions had a gel-like consistency that was resistant to flow. The magnitude of the ζ-potentials of the nanoemulsions and emulsions decreased as the peptide concentration was increased, especially for the fraction UP–10F (Figure 1, lines, right axis). Lee et al. [32] and Bouyer et al. [33] also reported that the ζ-potentials for WPI and β-lactoglobulin-stabilized (nano)emulsions decreased as protein concentration increased.

The UP–10F (nano)emulsions displayed a pH dependency at the 2 and 4% peptide concentrations. Fraction UP–10F failed to form a nanoemulsion at the 2 and 4% concentrations due to the proximity of the peptide solution pH (4.98–5.88) to the isoelectric point (pI) of WPI proteins (pH 4.00–5.50). Conformational changes around the pI facilitate peptide–peptide hydrophobic interactions [34], diminishing their interfacial activities in emulsions and causing droplets to flocculate and coalesce. The ζ-potential of the UP–10F peptide solution was −15.6 mV (1%), which diminished to −4.0 mV (4%). Thus, as the peptide concentration increased, the electrostatic repulsion between peptide molecules declined to favor peptide–peptide hydrophobic aggregation. The increased hydrophobic and reduced repulsive interactions between the peptides explain the gel-like emulsions evidenced at the 2% and 4% UP–10F peptide concentrations. Proteins form coagulum-type gel emulsions around their pI [34], supporting the current observation.

Conversely, the UC–10F nanoemulsion droplets were smallest at 2% but comparable at the 1 and 4% peptide concentrations (Figure 1, bars, left axis). The ζ-potentials of the peptide solution ranged from −12.1 mV (pH 7.00 at 1%) to −9.4 mV (pH 6.67 at 4%). The high net charge (increased intermolecular repulsions between the peptides), coupled with the remoteness of the pHs from the pI of WPI proteins, provided electrostatic charge stabilization of the droplets, irrespective of the peptide concentration. The magnitude of the ζ-potential of the UC–10F nanoemulsions decreased slightly as peptide concentration increased (Figure 1, lines, right axis), due to electrostatic charge screening resulting from the increasing ionic strength of the peptide solutions [32]. In agreement, the conductivity of the peptide solutions increased from 2.81 (1%) to 3.09 mS/cm (4%). Irrespective of the concentration, the ζ-potential remained relatively high for the UC–10F nanoemulsions, consistent with the electrostatic charge stabilization of nanoemulsions [16].

### 3.5. Effects of Storage Temperature and Time on (Nano)Emulsion Properties

Nanoemulsions must be stable against destabilization mechanisms during storage to permit their use in food and other applications.

#### 3.5.1. Droplet Size and ζ-Potential

**Figure 2:**
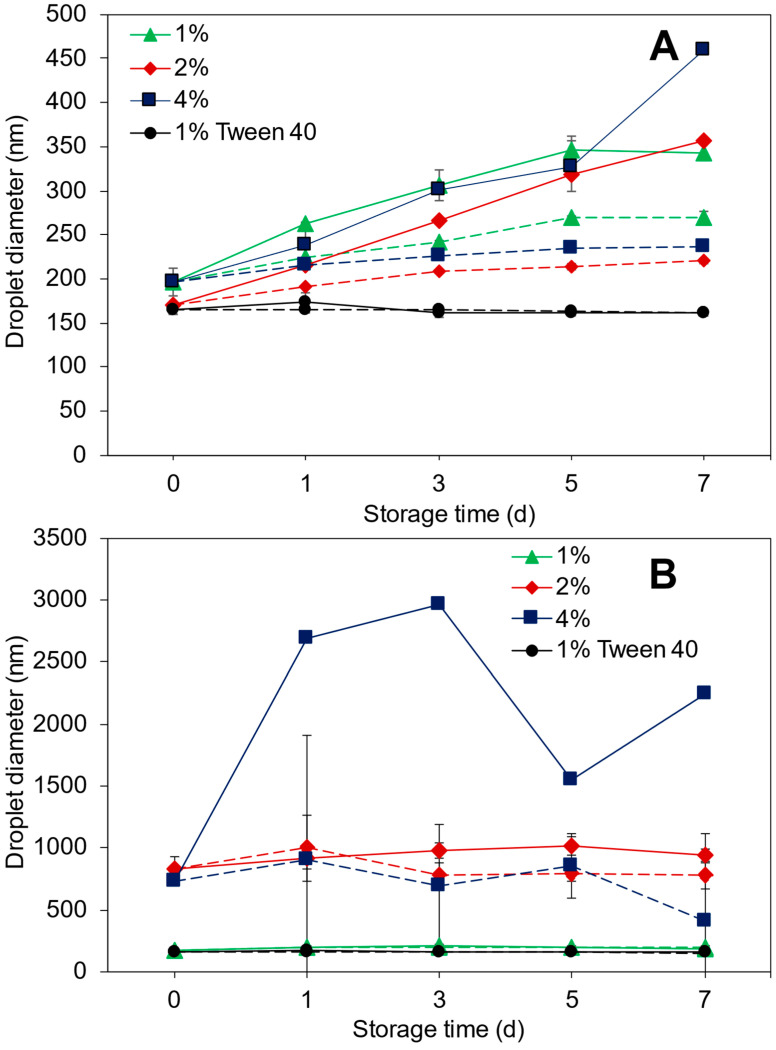
Storage temperature (4 and 25 °C) and time (0–7 d) on the droplet diameters of UC–10F chymotrypsin (**A**) and UP–10F pepsin (**B**) (nano)emulsions at varying emulsifier concentrations (1–4%). Legend: 4 °C, broken lines; 25 °C, solid lines; and 1% Tween 40, black lines.

Figure 2 shows the evolution in (nano)emulsion droplet size stored for seven (7) d at 4 or 25 °C. The droplet size (196 nm) increased for the 1% UC–10F nanoemulsion, which was more severe at 25 °C (344 nm) than at 4 °C (270 nm) (Figure 2A). For the 1% UP–10F nanoemulsion, the droplet size (174 nm) increased only slightly after 24 h (4 °C, 196 nm and 25 °C, 201 nm) and was unchanged for the remainder of the 7 d at 4 °C but decreased slightly at 25 °C (Figure 2B).

**Figure 3:**
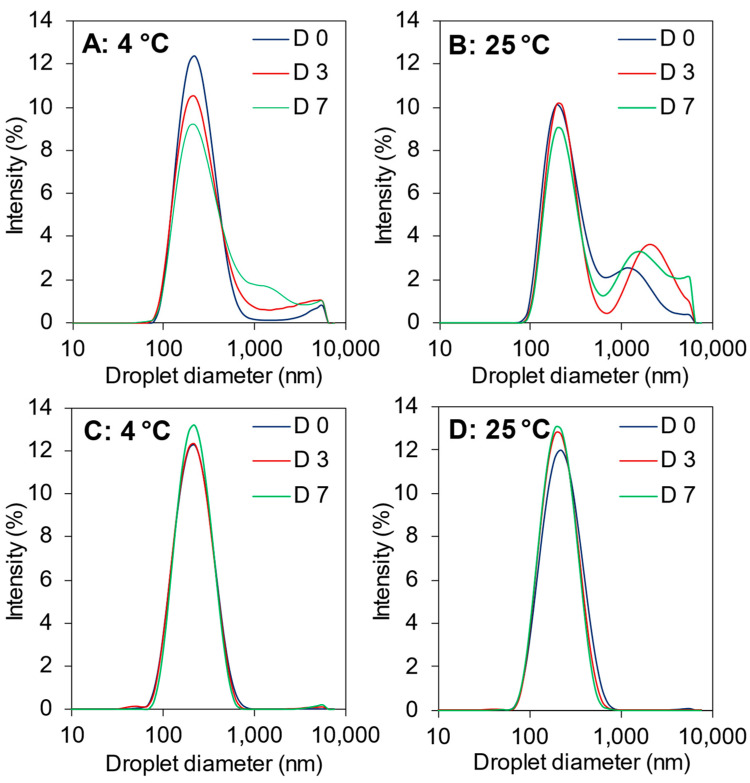
Droplet size distribution of UC–10F chymotrypsin (**A**,**B**) and UP–10F pepsin (**C**,**D**) WPI fraction nanoemulsions. Emulsifier concentration, 1%. Samples were stored at 4 and 25 °C for 7 d. D 0, D 3, and D 7 represent days 0, 3, and 7, respectively.

**Figure 4:**
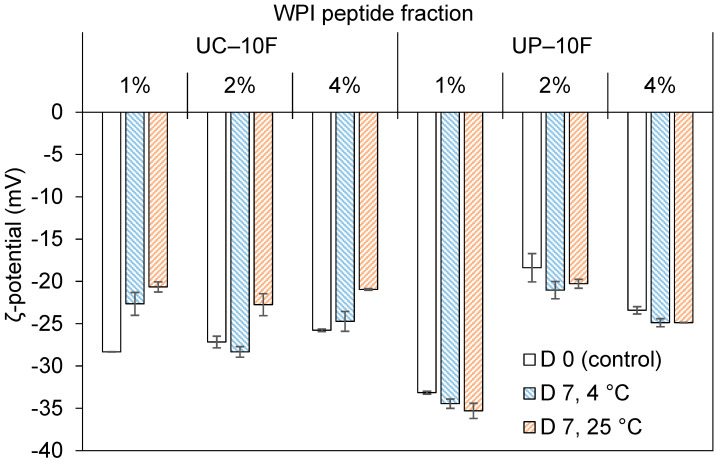
ζ-potential of nanoemulsions or emulsions formed with 1%, 2%, and 4% UC–10F chymotrypsin and UP–10F pepsin WPI fractions on D 0 (control) and 7. Samples were stored at 4 °C or 25 °C.

Likewise, the droplet size distribution became slightly bimodal for the 1% UC–10F nanoemulsion, especially at 25 °C but was unchanged for the 1% UP–10F nanoemulsion (Figure 3). Increasing the peptide concentration improved the stability of the UC–10F nanoemulsions, especially at 2%, stored at 4 °C (Figure 2A). The droplet size tended to decrease in the 2 and 4% UP–10F emulsions stored at 4 °C but increased at 25 °C (Figure 2B). The ζ-potential decreased (less negative) for the UC–10F nanoemulsions but increased slightly for the UP–10F (nano)emulsions during storage (Figure 4). Irrespective of the storage temperature, the coagulum gel emulsion formed at the 2 and 4% UP–10F concentration showed cracks with subsequent drainage of the aqueous phase (Supplementary Figure S3).

The stability of the 1% UP–10F nanoemulsion against droplet aggregation resulted from the formation of stronger and charged interfacial films (ζ-potentials = −33.2 to −33.8 mV (4 °C) and −35.9 mV (25 °C)), whereas the converse holds for the UC–10F nanoemulsion (ζ-potentials = −28.3 to −22.7 mV (4 °C) and −20.7 mV (25 °C)). Better stability for the UC–10F nanoemulsion at 4 °C than at 25 °C was due to: (i) more enhanced charge stabilization of the droplets at 4 °C than at 25 °C and (ii) increased viscosity in the nanoemulsions stored at 4 °C, due to stronger solvent–peptide interactions [35]. Generally, globular protein-stabilized emulsions exhibit better stability at lower storage temperatures than higher temperatures [36,37]. Moreover, the slight increase in the UC–10F nanoemulsion droplet size with storage was due to rearrangement and realignment of >10 kDa WPI peptides at the droplet interface [16].

Irrespective of the peptide fraction, enhanced stability occurred at lower peptide concentrations and storage temperature (Figure 2 and Figure 3). With increasing peptide concentration, droplet size growth depended on factors other than electrostatic effects (ζ-potentials remained relatively high). At higher peptide concentrations, not all peptides are adsorbed onto droplet interfaces. Hence, excess peptides in solution formed larger aggregates and caused droplets to flocculate by a depletion mechanism [38,39], which was more significant in (nano)emulsions stored at 25 °C. Secondly, higher peptide concentrations favored hydrophobic interactions between adsorbed peptide layers [40]. These hydrophobic interactions are enhanced during extended storage, increasing the effective surface hydrophobicity of the droplets, and causing droplets to flocculate [37,40]. As discussed in Section 3.2 (Table 1), fraction UP–10F had more hydrophobic peptide residues than fraction UC–10F. The substantial hydrophobic character, coupled with the proximity of the peptides to the pI of the WPI proteins of the UP–10F peptidic fraction, led to the coagulum gel emulsions and drainage observed in the 2 and 4% UP–10F emulsions (Supplementary Figure S3).

#### 3.5.2. Creaming Stability

Creaming in emulsions causes visible rings in beverage emulsions and leads to products with an uneven consistency, texture, and sensory characteristics [40]. The 1% and 2% UC–10F nanoemulsions stored at 25 °C creamed on D 3 (8%, reaching 12% by D 7) and 5 (8%, reaching 10% by D 7), respectively, but no creaming occurred in the nanoemulsions stored at 4 °C (Table 3). No creaming occurred in the 4% UC–10F and 1% UP–10F nanoemulsions. The creaming results were consistent with the droplet size and size distribution results (Figure 2 and Figure 3). Although the 4% UC–10F nanoemulsion stored at 25 °C exhibited larger droplet sizes than the 2% nanoemulsion (Figure 2A), it was stable against creaming due to a more rigid network and increased viscosity, resulting in an improved droplet packing [39].

### 3.6. Effect of Peptide Fractionation on Nanoemulsion Formation

**Figure 5:**
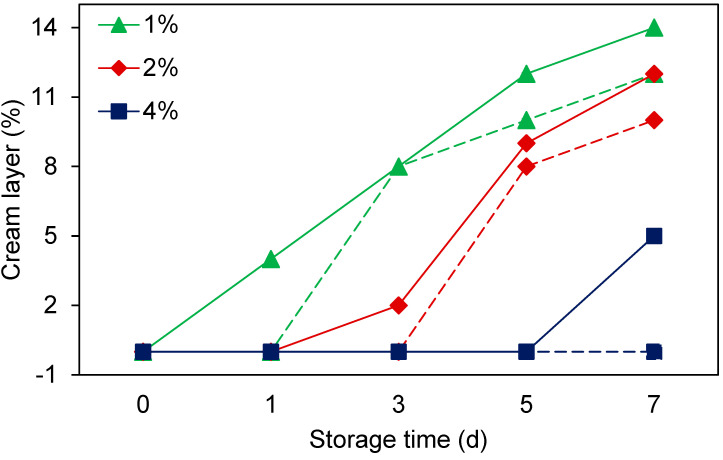
Influence of peptide fractionation on creaming stability in nanoemulsions during 7 d at 25 °C. Emulsifiers: UC–10F chymotrypsin WPI fraction (broken lines) and unfractionated chymotrypsin WPI hydrolysate (solid lines) at varying concentrations (1–4%). Data for unfractionated chymotrypsin WPI hydrolysate were from Adjonu et al. [1].

In a previous study, unfractionated WPI hydrolysate obtained by pepsin hydrolysis acted poorly as an emulsifier in a nanoemulsion [1]. In the current study, UF produced peptide fraction UP–10F from crude pepsin WPI hydrolysate, capable of forming nanoemulsions (diameter = 174 ± 2.28 nm) but at a lower peptide concentration only. Fraction UC–10F, from chymotrypsin WPI hydrolysis, formed nanoemulsions with smaller droplets (diameter = 196 ± 0.80 nm) than the unfractionated WPI hydrolysate (diameter = 288 ± 5.11 nm). The UC–10F nanoemulsions exhibited creaming at the 1 and 2% peptide concentrations only, but creaming occurred at all concentrations for the unfractionated WPI hydrolysate nanoemulsions, which escalated at the lower peptide concentrations (Figure 5). Studies comparing unfractionated hydrolysates with their 1–30 kDa (whey protein concentrate) and >10 kDa (WPI) fractions showed that fractionated peptides had better emulsifying activity than the unfractionated hydrolysates [35,41].

**Figure 6:**
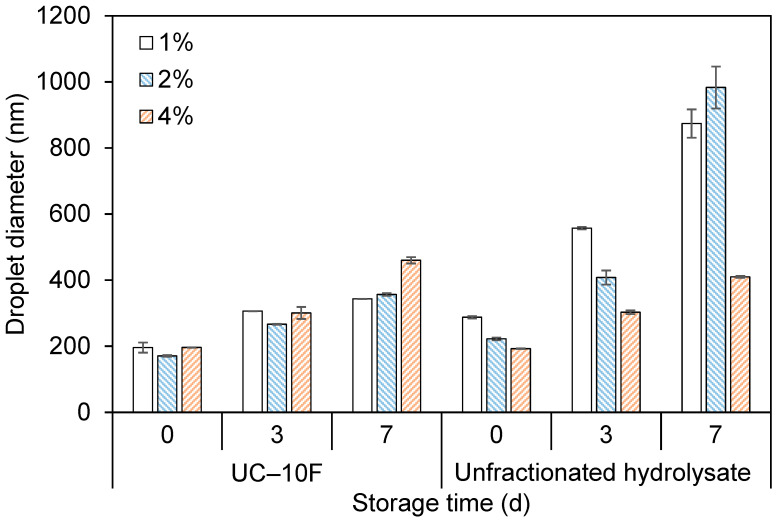
Influence of ultrafiltration on nanoemulsion stability stored at 25 °C for 7 d. Emulsifier types included UC–10F chymotrypsin WPI fraction and unfractionated chymotrypsin WPI hydrolysate, at varying concentrations (1–4%). Data for unfractionated chymotrypsin WPI hydrolysate were from Adjonu et al. [1].

The fractions UC–10F and UP–10F nanoemulsions were more stable against droplet size growth than the unfractionated WPI hydrolysate nanoemulsions, especially at lower peptide concentrations (<2%), but comparable stabilities existed at the higher peptide concentration (4%) (Figure 6). Thus, UF concentrated larger MW peptides in fractions UC–10F and UP–10F (22 to 46% of the total peptide yield recovered after UF; data are not shown), with a greater interfacial property than the unfractionated hydrolysates. Enriching larger and more surface-active peptides in fractions UC–10F and UP–10F diminished competitive adsorption between the smaller and poorly surface-active peptides (<10 kDa) and the larger peptides, resulting in the better interfacial activity of fractions UC–10F and UP–10F. Additionally, fractions UC–10F and UP–10F formed stronger interfacial films that were more resistant to fracture. In addition, the unfractionated WPI hydrolysates produced weak interfacial films due to a ‘patch-like’ adsorption of peptides onto the droplet interface, consisting of small and large peptides [42]. A weak interfacial film increases the rate of droplet flocculation, coalescence, and creaming [26,42] in nanoemulsions, especially at lower peptide concentrations (Figure 5 and Figure 6). However, the aqueous phase viscosity increased at the 4% peptide concentration [35] and enhanced stability, irrespective of UF treatment.

In conclusion, WPI-derived bioactive peptide fractions were successfully applied as emulsifiers in food-grade nanoemulsions with varying outcomes. Nanoemulsion formation and stability depended on the peptide size range, concentration, storage temperature and time, and the hydrolytic enzyme type. Peptide size determined the short (<12 h) and long (>24 h) term stability of nanoemulsions by influencing the peptides’ interfacial viscous effect and charge-stabilizing properties. The highly bioactive <10 kDa peptide fractions acted poorly as stabilizers in nanoemulsions, but the moderately bioactive >10 kDa fractions exhibited enhanced stability in nanoemulsions. Ultrafiltration enriched larger and more surface-active peptides in fractions UC–10F and UP–10F, possessing better functionality in nanoemulsions than unfractionated WPI hydrolysates. Thus, utilizing WPI and other food proteins’ bioactive peptides as emulsifiers in nanoemulsions provides an alternative to incorporating bioactive peptides as functional and nutraceutical ingredients in food systems. Thus, WPI peptides serve a dual function as bioactive compounds and emulsifiers in nanoemulsion. Further investigation is ongoing to enrich specific WPI peptide fractions with excellent bioactive and interfacial activities, capable of forming and stabilizing nanoemulsion droplets.

## Figures and Tables

**Table 1:** Bioactivity and surface hydrophobicity of WPI peptide fractions.

Peptide Fractions	Antioxidant Activity (µmol TE/mg peptide) *		ACE-Inhibition (IC_50_, mg peptide/mL) *		Surface Hydrophobicity	
	Chymotrypsin	Pepsin	Chymotrypsin	Pepsin	Chymotrypsin	Pepsin
1–3 kDa	0.30 ± 0.04 ^a^	0.25 ± 0.04 ^a^	0.565 ± 4.8 ^a^	0.38 ± 51.5 ^a^	≤44	
3–5 kDa	0.28 ± 0.04 ^a^	0.21 ± 0.04 ^ab^	1.041 ± 72.4 ^c^	0.97 ± 90.7 ^b^		
5–10 kDa	0.26 ± 0.05 ^a^	0.21 ± 0.02 ^ab^	0.872 ± 75.3 ^b^	1.24 ± 42.1 ^c^		
>10 kDa	0.24 ± 0.04 ^a^	0.14 ± 0.02 ^b^	1.119 ± 46.6 ^c^	1.90 ± 27.0 ^d^	1624.5 ± 4.0 ^b^	2088.5 ± 8.0 ^a^

* Different superscript letters in each column represent significant differences (*p* < 0.05) between means. IC_50_ = peptide concentration to inhibit 50% of angiotensin-I-converting enzyme (ACE) activity; TE = Trolox equivalent.

**Table 2:** Droplet diameter and ζ-potential of (nano)emulsions prepared with the WPI bioactive peptide fractions and Tween 40 at time 0 and 12 or 24 h.

Emulsifer Type (Concentration)	0 h		Storage Time (h) at 25 °C	Droplet Diameter (nm)	ζ-Potential (mV)
	Droplet Diameter (nm)	ζ-Potential (mV)			
Chymotrypsin WPI fractions					
1–3 kDa (2%)	218 ± 2.9	−35.9 ± 1.49	12	3125 ± 0.71	−32.8 ± 1.41
3–5 kDa (2%)	290 ± 6.7	−27.1 ± 1.61	12	1697 ± 236	−24.2 ± 0.50
5–10 kDa (2%)	199 ± 6.1	−29.2 ± 1.54	12	2794 ± 64	−19.4 ± 0.35
UC–10F (1%)	196 ± 0.8	−28.3 ± 0.04	24	262 ± 4.8	−25.2 ± 0.05
Pepsin WPI fractions					
1–3 kDa (2%)	4122 ± 632	−28.5 ± 0.39	12	290 ± 56	−29.3 ± 0.57
3–5 kDa (2%)	1233 ± 515	−19.5 ± 0.35	12	560 ± 280	−22.7 ± 0.53
5–10 kDa (2%)	4085 ± 1258	−15.4 ± 1.06	12	1741 ± 1122	−17.8 ± 2.02
UP–10F (1%)	174 ± 2.3	−33.2 ± 0.85	24	201 ± 9.4	−32.7 ± 1.85
Non-ionic surfactant					
Tween 40 (1%)	166 ± 5.5	−7.85 ± 0.20	24	173 ± 10.7	−8.52 ± 0.70

Note: Peptide concentrations were chosen based on their MW ranges.

**Table 3:** Creaming (%) in UC–10F WPI fraction nanoemulsions at 1 and 2% peptide concentrations.

Storage Time (d)	1 (%)		2 (%)	
	4 °C	25 °C	4 °C	25 °C
0	–	–	–	–
1	–	–	–	–
3	–	8	–	–
5	–	10	–	8
7	–	12	–	10

Note: – indicates no creaming in the nanoemulsions. UC–10F = chymotrypsin WPI fraction.

## Data Availability

The data presented in this study are available on request from the corresponding author. The data are not publicly available due to privacy.

## Supplementary Materials

The following supporting information can be downloaded at: https://www.mdpi.com/article/10.3390/foods11121812/s1, Figure S1: Appearance at time 0 and 12 h of the 1–3, 3–5, and 5–10 kDa WPI fraction (nano)emulsions. Emulsions were prepared with 2% peptide emulsifier and stored at 25 °C; Figure S2: Illustration of the stabilization and destabilization of nanoemulsions produced by different WPI bioactive peptide fractions. WPI bioactive peptide fractions were obtained by chymotrypsin hydrolysis; Figure S3: After 24 h, the 4% UP–10F pepsin WPI fraction emulsion showed a solid gel-like matrix with cracks and subsequent drainage of the aqueous phase.
