# Supplementary material for: Whey Protein Peptides Have Dual Functions: Bioactivity and Emulsifiers in Oil-In-Water Nanoemulsion
Source: Foods. 2022 Jun 20;11(12):1812. doi: 10.3390/foods11121812 (PMC9222674; doi:10.3390/foods11121812)
Supplement: Supplementary file 1 [file foods-11-01812-s001.zip › Supplementary Figure S1.pdf]

Figure S1

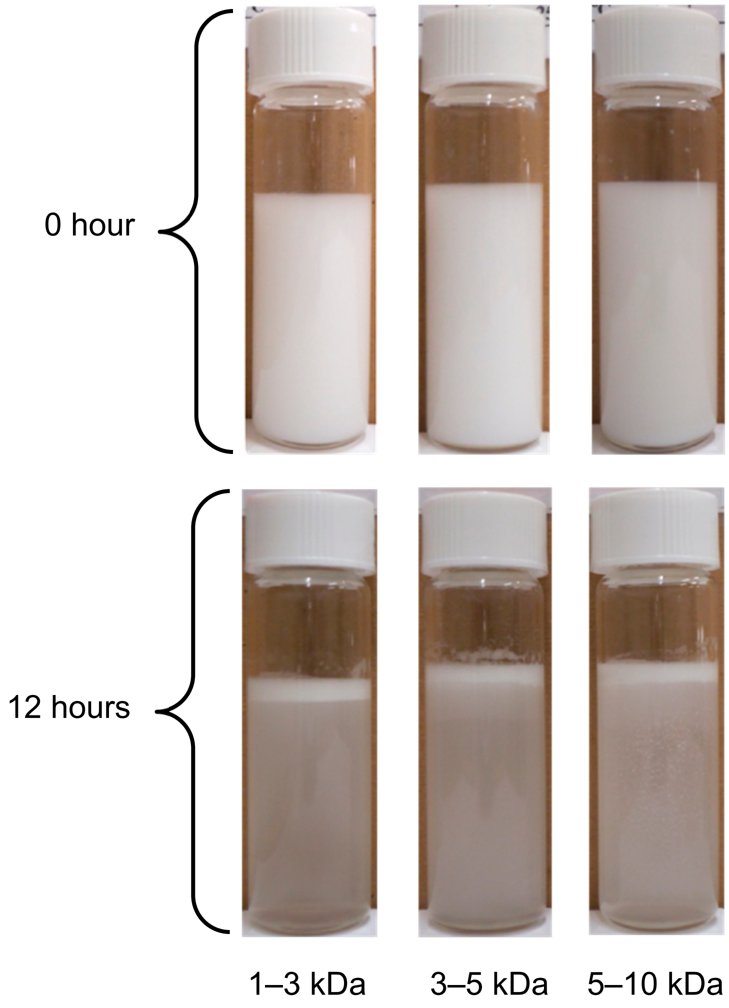

Figure S1: Appearance at time 0 and 12 hours of the 1–3, 3–5, and 5–10 kDa WPI fraction (nano)emulsions. Emulsions were prepared with 2% peptide emulsifier and stored at 25 °C.
