# Supplementary material for: Whey Protein Peptides Have Dual Functions: Bioactivity and Emulsifiers in Oil-In-Water Nanoemulsion
Source: Foods. 2022 Jun 20;11(12):1812. doi: 10.3390/foods11121812 (PMC9222674; doi:10.3390/foods11121812)
Supplement: Supplementary file 1 [file foods-11-01812-s001.zip › Supplementary Figure S2.pdf]

Figure S2

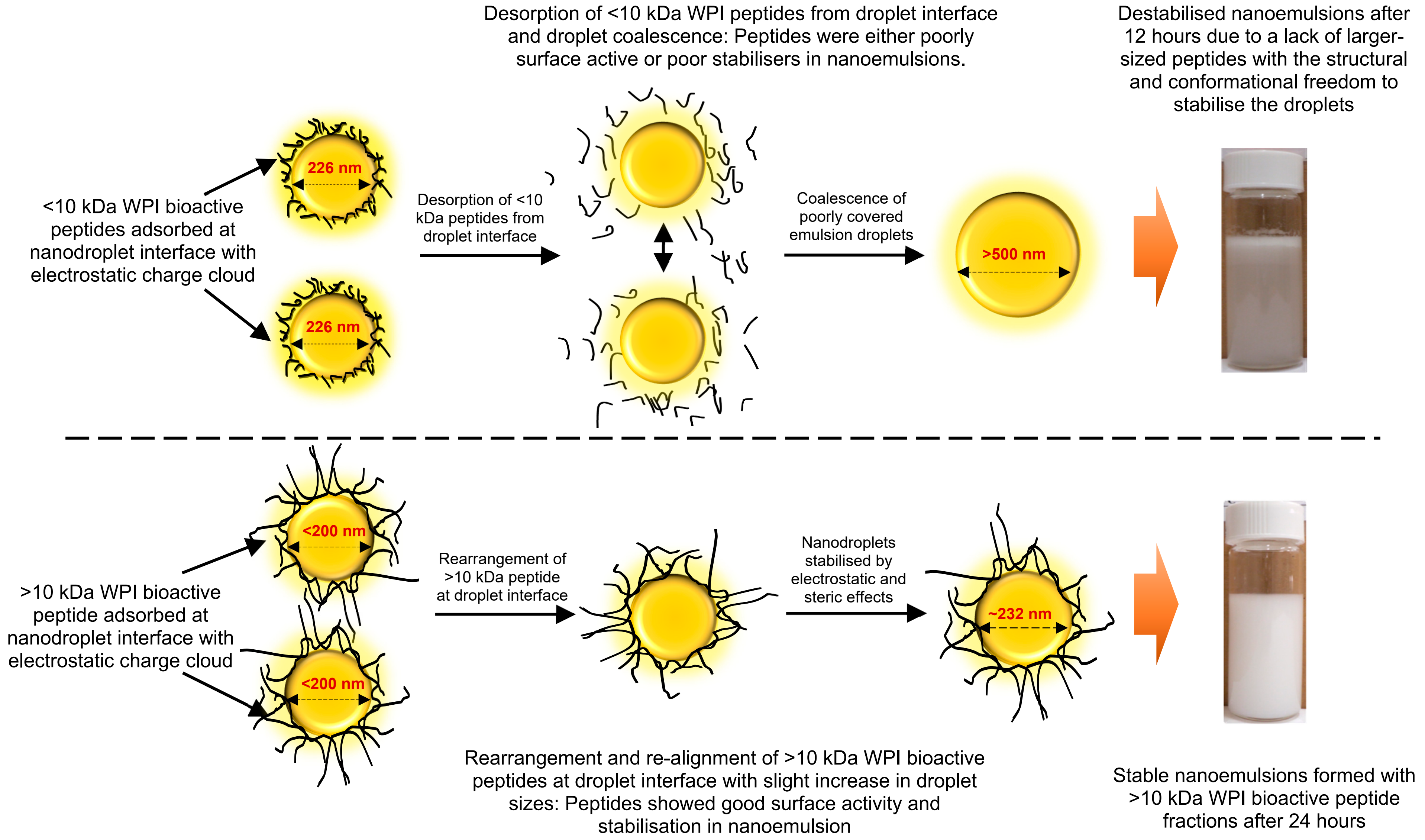

Figure S2: Illustration of the stabilization and destabilization of nanoemulsions produced by different WPI bioactive peptide fractions. WPI bioactive peptide fractions were obtained by chymotrypsin hydrolysis.
