# Supplementary material for: Whey Protein Peptides Have Dual Functions: Bioactivity and Emulsifiers in Oil-In-Water Nanoemulsion
Source: Foods. 2022 Jun 20;11(12):1812. doi: 10.3390/foods11121812 (PMC9222674; doi:10.3390/foods11121812)
Supplement: Supplementary file 1 [file foods-11-01812-s001.zip › Supplementary Figure S3.pdf]

Figure S3

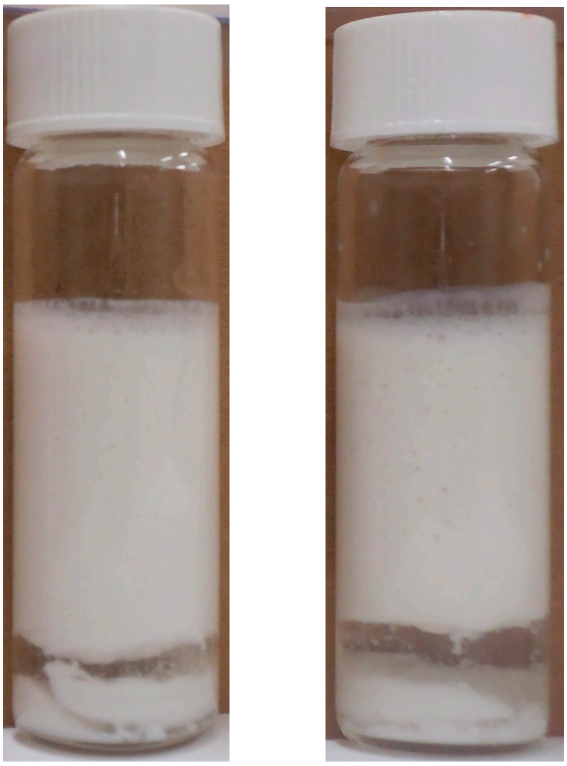

Stored at 4 ^{o}C Stored at 25 ^{o}C

Figure S3: After 24 hours, the 4% UP–10F pepsin WPI fraction emulsion showed a solid gel-like matrix with cracks and subsequent drainage of the aqueous phase.
